# Antioxidant potential, antimicrobial activity, polyphenol profile analysis, and cytotoxicity against breast cancer cell lines of hydro-ethanolic extracts of leaves of (*Ficus carica* L.) from Eastern Morocco

**DOI:** 10.3389/fchem.2024.1505473

**Published:** 2024-11-27

**Authors:** Aziz Tikent, Salah Laaraj, Toufik Bouddine, Mohamed Chebaibi, Mohamed Bouhrim, Kaoutar Elfazazi, Ali S. Alqahtani, Omar M. Noman, Lhoussain Hajji, Larbi Rhazi, Ahmed Elamrani, Mohamed Addi

**Affiliations:** ^1^ Laboratoire d’Amélioration des Productions agricoles, Biotechnologie and Environnement (LAPABE), Faculté des Sciences, Université Mohammed Premier, Oujda, Morocco; ^2^ Regional Center of Agricultural Research of Tadla, National Institute of Agricultural Research (INRA), Rabat, Morocco; ^3^ Laboratory of Environmental, Ecological and Agro-Industrial Engineering (LGEEAI), Faculty of Science and Technology (FST), Beni Mellal, Morocco; ^4^ Bioactive and Environmental Health Laboratory, Moulay Ismail University of Meknes, Meknes, Morocco; ^5^ Ministry of Health and Social Protection, Higher Institute of Nursing Professions and Health Techniques, Fez, Morocco; ^6^ Biomedical and Translational Research Laboratory, Faculty of Medicine and Pharmacy of Fez, Sidi Mohamed Ben Abdellah University, Fez, Morocco; ^7^ Biological Engineering Laboratory, Faculty of Sciences and Techniques, Sultan Moulay Slimane University, Beni Mellal, Morocco; ^8^ Department of Pharmacognosy, College of Pharmacy, King Saud University, Riyadh, Saudi Arabia; ^9^ Institut Polytechnique UniLaSalle, Université d'Artois, Beauvais Cédex, France

**Keywords:** Eastern Morocco, fig leaf, antioxidant, antimicrobial, anticancer

## Abstract

**Introduction:**

Many beneficial compounds found in fig leaves can be used in tea and medicine. These compounds aid with digestion, reduce inflammation, and treat diabetes and bronchitis. Chetoui, Malha, Ghoudane, and Onk Hmam fig leaf hydro-ethanol extracts from Eastern Morocco were analyzed for metabolites and biological activities.

**Methods, results, and discussion:**

HPLC-UV examination revealed that the leaf extract included mainly caffeine, rutin, and ferrulic acid. Spectrophometric results show that Malha leaf is rich in polyphenols (62.6 ± 1.3 mg GAE/g) and flavonoids (26.2 ± 0.1 mg QE/g). Chetoui leaf contains the highest vitamin C content (8.2 ± 0.1 mg Asc A/100 g DW), while Onk Hmam leaf has the highest condensed tannin (4.9 ± 0.1 mg CatE/g). The investigations found that all leaf extracts were antioxidant-rich, with strong Pearson bivariate correlation between bioactive polyphenol levels and antioxidant tests for DPPH, β-carotene, ABTS, and TAC (values of −0.93, −0.94, −0.85, and 0.98, respectively). The coefficients for flavonoid content were −0.89, −0.89, −0.97, and 0.80, respectively. Disk diffusion and MIC results show that the hydro-ethanol fig leaf extracts eliminate fungi and bacteria. In addition, these fig leaf extracts showed promise cytotoxicity against the breast cancer cell lines MCF-7, MDA-MB-231, and MDA-MB-436 and an interesting selectivity index. In silico leaf bioactive component analysis revealed that myricitin inhibited NADPH oxidase the greatest (gscore −6.59 Kcal/mol). Trans-ferulic acid inhibits Escherichia coli beta-ketoacyl-[acyl carrier protein] synthase (−6.55 kcal/mol), whereas quercetin inhibits *Staphylococcus aureus* nucleoside diphosphate kinase (−8.99). CYP51 from *Candida albicans* is best treated with kaempferol and myricitin. Both had a glide gscore of −7.84 kcal/mol. Rutin has the most potent Sespace 3 anticancer activity, with a glide gscore of −7.09 kcal/mol.

**Conclusion:**

This research indicates that fig leaf extracts from the region can be used in medicine, food, natural cosmetics, and breast cancer prevention. To maximize the value of these leaves, their use must be carefully studied. Naturally, this fortunate tree’s diversity must be preserved and enhanced.

## 1 Introduction

Oxidative stress occurs when a species’ reactive and antioxidant defenses are not balanced. This condition is linked to numerous chronic diseases, including cancer, diabetes, neurodegenerative diseases, and cardiovascular diseases (McCord, 2000; Vladimir-Knežević et al., 2011). In addition to their physiological functions as typical secondary metabolites in plants, phenolic compounds possess advantageous health benefits as antioxidants (Çalişkan and Polat, 2011). Researchers have conducted thorough investigations on natural antioxidant molecules due to their potential in treating disorders associated with oxidative stress. Additionally, they are employed in the food industry due to their biological characteristics, particularly as antimicrobials (Taviano et al., 2013). Antioxidants can improve immune function and help prevent diseases such as cancer, cardiovascular disease, macular degeneration, cataracts, and asthma. They defend the body against the harmful effects of free radicals, which are produced as byproducts of regular metabolism (Sadia et al., 2014). The emergence of microbial resistance to existing antibiotics, along with the adverse effects caused by modern drugs and the scarcity of antibiotics in development, has underscored the importance of finding new antibacterial agents. Consequently, researchers have turned their attention to investigating the antimicrobial properties of medicinal plants (Mulat, Pandita and Khan, 2019; Reddy et al., 2022). One of the intriguing biological characteristics of polyphenols is their ability to prevent cancer. There is much epidemiological research indicating that consuming a diet abundant in fruits and vegetables may decrease the likelihood of developing specific types of malignancies. In part, the impact has been attributed to inherent polyphenols. Indeed, multiple laboratory experiments conducted both outside of and within a living organism have shown that natural compounds called polyphenols have the potential to be used in cancer prevention and treatment (Stagos et al., 2012; Zhou et al., 2016).

The fig tree, scientifically known as *Ficus carica* L., is a member of the Moraceae family (Nuri and Uddin, 2021). It is a fruit tree from classical antiquity that is associated with the origins of horticulture in the Mediterranean basin (Aradhya et al., 2010). In spite of the fact that it is a perennial plant, the fruit, leaf, and latex of the fig are the primary products that are developed and utilized in the market (Badgujar et al., 2014; Palmeira et al., 2019). The leaf has been utilized extensively for therapeutic purposes, and the decoction of the leaves is currently being taken as a beverage (Morovati et al., 2022; Yang et al., 2023). Fig leaves, a by-product of fig fruit production, can serve as a vital source of bioactive chemicals for many industries. Potential uses include replacing synthetic chemicals with natural alternatives in the food sector, thus enhancing the physical and sensory properties of the foods in which they are incorporated (Chamorro et al., 2022). The fig leaf contains tocopherol and phenolic chemicals, which are powerful antioxidants (Konyalιoğlu et al., 2005). The fruits, roots, and leaves of the fig tree have been utilized in traditional medicine to address numerous illnesses such as diarrhea, indigestion, sore throats, coughs, bronchial problems, inflammatory and circulatory disorders, ulcerative illnesses, and malignancies (Gilani et al., 2008; Lee and Cha, 2010). The integration of chemical structures from various drugs into plant derivatives justifies the clear rebound in plant-based medicine utilization. Traditional medicine is the primary healthcare method used by 80% of the world’s population. Most traditional medicines involve the use of plant extracts or their active components (Payyappallimana, 2010).

There are many bioactive compounds found in *F. carica*, such as phenolic compounds, phytosterols, organic acids, anthocyanin composition, triterpenoids, coumarins, and volatile compounds such as hydrocarbons, aliphatic alcohols, and a few other secondary metabolites that come from different parts of the plant (Kalefa and Al-Shawi, 2023; Tkachenko et al., 2017). The aqueous extract of *F. carica* leaves contained phenolic acids such as 3-O- and 5-O-caffeoylquinic acids, ferulic acid, quercetin-3-O-glucoside, quercetin-3-O-rutinoside, and organic acids (oxalic, citric, malic, quinic, shikimic, and fumaric acids) (Oliveira et al., 2009). Chunyan et al. (2008) (Chunyan et al., 2008) discovered that psoralen and bergapten are furocoumarins in F. *carica* leaves using countercurrent chromatography, 1H NMR proton magnetic and nuclear resonance, 13C NMR carbon, and mass spectrometry. Previous studies have identified other coumarins in F. *carica* leaves, such as bergapten, 4′, 5′dihydropsoralen (marmesin), and umbelliferone (Innocenti, Bettero and Caporale, 1982). Vaya and Mahmood (Vaya and Mahmood, 2006) used high-performance liquid chromatography and mass spectrometry to study *F. carica* leaves and found that the main flavonoids that were present were quercetin and luteolin. Both of these flavonoids are important in the body because they fight free radicals, prevent inflammation, keep the immune system in check, and help prevent cancer (Lansky et al., 2005; Zand et al., 2000). Teixeira et al. (2006) identified many phenolic compounds in F. *carica* leaves, including chlorogenic acid and rutin. Rutin, also called rutoside, is a diglucoside of quercetin, a potent antioxidant. It is also the most widely studied flavonol in terms of pharmacology (Teixeira et al., 2006). Isocoumarins, isomers of flavonoid phenolic compounds, are recognized for their antioxidant, antimicrobial, and anticancer properties. For instance, 3-aryl isocoumarins are isomers of myricitin, kaempferol, and quercetin (Sudarshan et al., 2015). The same applies to the 3-glycosyl isocoumarins, which are isomers of rutin and naringin (Sudarshan and Aidhen, 2017). The number and content of bioactive chemicals vary by genotype, latitude, geographical region, and leaf collection period (Oliveira et al., 2010). It has been generally established that aqueous or acetone is suitable for extracting greater molecular weight flavanols, whereas methanol is more successful for extracting lower molecular weight polyphenols. Ethanol is selected due to its reputation as a safe substance for human consumption and its efficacy as a solvent for extracting polyphenols (Do et al., 2014).

In Morocco’s Eastern region, the fig tree is a prominent fruit tree. This is exemplified by the annual Aghbal Fig Festival in Ahfir, the region’s primary fig producer, which honors the fig fruit. This event recognizes the economic and socio-cultural importance of figs and their products. While fig fruits are well-known for their nutritional benefits and flavor, fig leaves are less so. As a result, it is critical to initially clarify the bioactive compound composition of hydro-ethanol fig leaf extracts. The purpose of this study is to demonstrate that fig leaves have the potential to be a valuable source of particular chemicals for application in food and phytopharmaceuticals. We want to increase the use and value of these leaves, while also boosting their reputation. To achieve the above, work was done to study the amounts of total polyphenols, flavonoids, condensed tannins, and vitamin C using spectroscopic methods. The extracts were also analyzed using high-performance liquid chromatography (HPLC) to determine the quality profile of the phenolic compounds that contribute to their bioactive activities. To test antioxidant capability, we used total antioxidant capacity, beta-carotene bleaching assay, DPPH scavenging, and ABTS scavenging. Furthermore, linear correlations are established to demonstrate the involvement of bioactive chemicals in antioxidant activity. Finally, we evaluate the extracts’ antimicrobial activity against five specific strains and their cytotoxicity against three breast cancer cell lines. The *in silico* method was employed to validate and corroborate the involvement of the biologically active compound in the examined biological activities. By looking at specific examples, the study aims to explain how certain bioactive compounds in the fig leaf extract show anticancer, antimicrobial, and antioxidant properties.

## 2 Materials and methods

### 2.1 Materials sampling

In June 2023, the leaves of four different varieties of fig trees (*F. carica* L.) were gathered in three distinct locations in Ahfir province, Eastern Morocco. Three of the examined varieties are uniferous: Chetoui (CH), Malha (MA), and Onk Hmam (OH). Meanwhile, Ghoudane (GD) is biferous. The voucher specimen of the plant is deposited at the plant section of Herbarium University Mohammed Premier Oujda Morocco (HUMPO 100). Leaf samples were collected at random, and an attempt was made to remove the influence of exposure by collecting the same number of leaves from all cardinal points: north, south, east, and west, as well as within the tree. For each variety, samples of 150 disease-free leaves (ten per tree and five trees per variety in each location) were picked, selected, and harvested. To safeguard the heat- and light-sensitive molecules, the leaves were dried for 20 days in an airy, dark, and room-temperature environment. To increase the contact surface between the sample and the extraction solvent, the dried leaves were ground into a fine, uniform powder that was then stored away from humidity.

### 2.2 Extraction process

A solid-liquid extraction technique was employed to extract antioxidant components from powdered fig leaves. A solvent containing 90% ethanol (v/v). The sample was extracted in the solvent using a fixed solid/liquid ratio of 1:10 (weight in grams/volume in milliliters). Subsequently, the concoction was agitated at a temperature of 50°C for duration of 90 min using a shaking water bath. For 20 min, the sample was centrifuged at 10,000 revolutions per minute at a temperature of 4°C. After centrifugation, it was filtered using a 0.45 μm polytetrafluoroethylene (PTFE) membrane filter. The aliquot extract was diluted to a final amount of 15 mL. The liquid extracts of fig leaves were promptly examined to determine the levels of the bioactive compounds being researched, as well as their antioxidant capabilities (Nakilcioğlu-Taş and Ötleş, 2021).

### 2.3 Bioactive compounds measurements

The Folin-Ciocalteu (FC) method was used to determine the total polyphenol content (TPC) (Tikent et al., 2023). 100 μL extract mixed with 500 µL FC reagent and 400 µL of 7.5% (w/v) Na_2_CO_3_. The mixture is stirred and incubated in the dark at room temperature for 10 minutes, and then the absorbance is measured at 760 nm. The results are reported in mg gallic acid equivalent (GAE) per gram of dry plant, with the gallic acid calibration curve as a reference. We used the AlCl_3_ method (Dehpour et al., 2009), to find out the total flavonoid content (TFC). To do the test, we mixed 500 µL of each extract with 1,500 µL of 95% methanol, 100 µL of 10% (m/v) AlCl_3_, 100 µL of 1 M sodium acetate, and 2.8 mL of distilled water. For 30 min, the mixture is mixed and incubated in the dark at room temperature. The blank is formed by replacing the extract with 95% methanol, and the absorbance at 415 nm is measured. With reference to the quercetin calibration curve, the results are presented in quercetin equivalent (QE) mg/g dry plant. The vanillin method was employed to determine the condensed tannin content (CTC) in an acidic medium (Mohti et al., 2020). The vanillin reagent was prepared by mixing equal volumes of: 8% (v/v) HCl, 37% (v/v) methanol, and 4% vanillin in methanol (m/v). Prior to the assay, the mixture was stored at 30°C. 200 μL of each extract to be analyzed was added to 1,000 µL of vanillin reagent; the mixture was shaken and then incubated in the dark for 20 min. Absorbance was measured at 500 nm against a blank consisting of a mixture of methanol (37%) and HCl (8%). The results are expressed as mg catechol equivalent (Cat E)/g dry plant matter using the catechol calibration curve. The 2,6-dichlorophenolindophenol (DCPIP) spectrophotometric method (Hadini et al., 2022) was used to determine the vitamin C content. We add 1 g of dried leaf powder to 10 mL of oxalic acid (1%) and stir for 15 min. Then, we mix 3 mL of the filtrate with 1 mL of DCPIP (5 mM) and measure the absorbance at 515 nm after 15 s. The concentration of ascorbic acid (Asc A) is measured in milligrams (mg) per 100 g of dry matter.

### 2.4 Antioxidant activity assessment

The antioxidant activity of the hydro-ethanolic extracts of the examined fig leaves at a concentration of 0.1 g/mL was determined using four separate tests. All experiments were performed in triplicate.

#### 2.4.1 2,2-diphenyl-1-picrylhydrazil (DPPH) free radical scavenging assay

The hydro-ethanol leaf extracts’ free radical scavenging ability was measured using the procedures given in references (Tikent et al., 2023; Zrouri et al., 2021). DPPH solution was prepared by solubilizing 2 mg of DPPH in 100 mL of methanol. Different concentrations ranging from 5 to 500 μg/mL were prepared. Afterwards, each concentration was added to 2.5 mL of the prepared DPPH methanol solution to the final volume of 3 mL. After 30 min of incubation at room temperature, the absorbance was measured at 515 nm against a blank. The DPPH free radical scavenging activity was estimated in percentage (%) using the following formula:

$$\text{Radical scavenging activity }\left(\%\right)=\left[\left(\left({\left[A\right]}_{\text{blank}}\;–{\left[A\right]}_{\text{sample}}\right)/{\left[A\right]}_{\;\text{blank}}\right)\right]\times 100$$

A _blank_ is the absorbance of the control reaction and A _sample_ is the absorbance of the extract at different concentrations. IC_50_ values were determined graphically from the curve of antioxidant concentration (mg/mL) versus % inhibition, generated by GraphPad Prism 8. Ascorbic acid was employed as a positive control.

#### 2.4.2 β-carotene bleaching assay

The antioxidant activity was performed using bleaching of a β-Carotene assay using the procedures given in the references (Elbouzidi et al., 2023; Rădulescu et al., 2021). First, 2 mg of β-carotene was dissolved in 10 mL of chloroform, and then mixed with 20 mg of linoleic acid and 200 mg of Tween-80. A vigorous stir was used to add 100 mL of distilled water to the flask after rotavapor at 40°C removed the chloroform mixture. A 96-well plate with triplicate samples was then incubated at 25°C for 30 min in the dark. Then, absorbance was measured spectrophotometrically at 470 nm immediately after hydro-ethanol fig leaf solution addition (t_0_) and 2 h later (t_1_) against a white reading containing all solution components but no-carotene. Butylated hydroxyanisole (BHA) was used as a standard reference.

$$\text{Residual color }\left(\%\right)=\left[\left(\left(\text{OD }t_{0}–\text{OD }t_{1}\right)/\;\left(\text{OD}t_{0}\right)\right)\right]\times 100$$

where ODt_0_ and ODt_1_ are the absorbance at time zero (t_0_) of Sample or Standard and ODt_1_ after 2 h (t_1_) respectively.

#### 2.4.3 ABTS scavenging activity assay

The scavenging capacity to the 2,2-Azino-bis-(3-ethylbenzothiazoline-6-sulfonic acid) (ABTS) radical of the hydro-ethanol fig Leaf extracts were investigated, as described by Nakyai et al. (2021) (Nakyai et al., 2021),with certain modifications. In order to generate ABTS⋅+, the ABTS solution was mixed with 2.45 mM potassium persulfate and left to incubate at room temperature for a period of 16–18 h in the absence of light. The solution was subsequently mixed with ethanol until it reached an absorbance of 0.70 ± 0.02 at a wavelength of 750 nm. A concentrated solution of extract was prepared in ethanol. L-ascorbic acid was used as a positive control. The ABTS assay was conducted by mixing 200 µL of diluted ABTS⋅+ solution with 20 µL of the test material. The reaction mixture was kept in the absence of light at ambient temperature for duration of 10 min, after which it was quantified using a microplate reader at a wavelength of 734 nm. A percentage of ABTS radical cation decolorizing activity was determined, similar to the DPPH assay.

#### 2.4.4 Total antioxidant capacity

The total antioxidant capacity was assessed using the phosphor-molybdenum method, as outlined in references (Chaudhary et al., 2015; Ouahabi et al., 2023; Taibi et al., 2023). The standard/extract solution, measuring 0.1 mL, was mixed with a reagent solution containing 0.6 M sulfuric acid, 28 mM sodium phosphate, and 4 mM ammonium molybdate. The mixture was then incubated at a temperature of 95°C for duration of 90 min. The solution was allowed to cool to the temperature of the surrounding environment, and the level of light absorption at a wavelength of 695 nm was determined. With the exception of the test sample, the blank solution included all of the reagents. A standard curve was generated utilizing ascorbic acid. The results were expressed in terms of ascorbic acid equivalents (Prieto et al., 1999).

### 2.5 HPLC-UV analysis

The phenolic composition of fig leaf extracts (0.1 g/mL) was analyzed chromatographically using an EC NUCLEOSIL column (5 μm, C18, 100–5,250 mm × 4.6 mm; Macherey-Nagel, Germany) in accordance with the procedures outlined in the protocol described by Kachmar et al. (2019), with minor adjustments. Water-phosphoric acid (0.01%) (A) and acetonitrile (B) comprised the mobile phase. A gradient was employed to obtain the following concentrations: 5% B after 3 min, 25% B after 13 min, 30% B at 25 min, 35% B at 35 min, 45% B at 39 min, 30% B at 25 min, 45% at 42 min, 55% B at 47 min, 75% at 56 min, 100% B at 60 min, 100% at 65 min, 5% at 73 min, and 5% at 80 min. UV detection at 280 nm was conducted. 20 μL was the volume of the injection, while the discharge rate was 0.9 mL/min. The apparatus for HPLC analysis is managed by the JASCO Chrom NAV2.0 HPLC software. The process of identification involved the comparison of the retention time of every pic to that of the corresponding standard.

### 2.6 Antimicrobial activities

For this experiment, a group of five microorganisms was used. There were two types of Gram-negative bacteria in the group, *Escherichia coli (ATCC 25922) and Pseudomonas aeruginosa (ATCC 27853)*, two types of Gram-positive bacteria, *Staphylococcus aureus (ATCC 25923) and Bacillus subtilis subsp. spizenii (ATCC 6633)*, and one type of pure fungus, *Candida albicans*. To support the growth of these bacteria, they were cultured on Biorad’s Muller-Hinton agar (MH) at a temperature of +4°C. To facilitate growth, the cultures were periodically transferred every fortnight and scrutinized for purity. The samples were solubilized in dimethyl sulfoxide (DMSO). Twenty milliliters of aseptic Mueller Hinton agar (Sigma, Paris, France) were added to Petri dishes and subsequently inoculated with a 200 μL cell suspension. The concentration of the cell suspension was adjusted to 10^6^ colony-forming units per milliliter (CFU/mL) using the McFarland 0.5 technique. Filter paper discs, devoid of bacteria or other microorganisms and with a diameter of 6 mm, were saturated with 20 μL of the extract solution (5 mg/disc). Subsequently, these discs were placed on the agar surface. The plates were incubated at a temperature of 37°C for a period of 24 h. The experimental group used gentamicin (15 µg/disc) and Fluconazole (60 µg/disc) as positive controls. The negative controls comprised paper discs holding 20 μL of DMSO or distilled water. The inhibitory zones were measured using digital calipers, and the measurements were repeated three times to minimize errors. A diameter of 14 mm or more for the inhibition zone, including the disc diameter, indicates a significant level of antibacterial activity (Philip et al., 2009). A serial dilution method was used in 96-well microtiter plates to find the minimum inhibitory concentration (MIC) for microbial growth. We found out how much extract to use in the test by letting the solvent evaporate from 1 mL of extract, mixing the dry extract with 20% v/v DMSO, and then adding 10 times as much Mueller-Hinton broth to it. After that, 100 μL of the bacterial or fungus solutions and dilutions were put into microtiter plates and left to grow at 37°C for 24 h. The positive control consisted of 100 μL of bacterium solution combined with 100 μL of Mueller Hinton broth. In contrast, the negative control consisted of 100 μL of diluent mixed with 100 μL of extract without bacteria. After 24 h, the positive and negative findings were assessed in relation to turbidity compared to the control well. The MIC values were determined as the minimum concentration of extract (from 16 to 1,000 μg/mL) that completely suppressed microbial development, as evidenced by a clear well,extracts were categorized as highly active when MICs were below 100 μg/mL, moderately active when values ranged from 100 to 625 μg/mL, and weakly active when values exceeded 625 μg/mL (Chusri et al., 2014). The validity of all extracts was assessed by conducting three separate tests (Tikent et al., 2023).

### 2.7 Cytotoxicity against breast cancer cell lines

#### 2.7.1 Cell culture

The study used two types of breast cancer cells: MCF-7 cells, which are estrogen receptor-positive, and MDA-MB-231 and MDA-MB-436 cells, which are estrogen receptor-negative. To keep the cells alive, they were kept in Dulbecco’s modified Eagle’s medium (DMEM) with 10% Fetal bovine serum (FBS) and 50 μg/mL gentamicin at 37°C with 5% CO_2_ in a humid room. The cells were cultured in 25-cm^2^ tissue culture flasks to maintain continuous growth. To investigate cell viability, the study used cells in the rapid expansion phase.

#### 2.7.2 Cell viability by MTT assay

To determine whether the studied extracts could inhibit the proliferation of cancer cells, we have used the 3-(4,5-dimethylthiazo-l-2-yl)-2,5-diphenyltetrazolium bromide (MTT) assay, following the method described in references (Chaudhary et al., 2015; Elbouzidi et al., 2023). MCF-7, MDA-MB-231, and MDA-MB-436 cells that were growing exponentially were put in 96-well plates at a density of 10^4^ cells per well in 100 µL of medium and left there for 24 h. To obtain various concentrations, the studied extracts were solubilized in 0.1% DMSO and serially diluted with medium. Different concentrations of the studied extracts (from 6.25 to 300 μg/mL) were prepared by dissolving them in 0.1% DMSO and diluting them with medium and cisplatin was used as a positive control at various concentrations ranging from 0.39 to 50 µg/mL. The cells were then exposed to varying concentrations of the studied extracts for 72 h. The control group cells were only given medium containing 0.1% DMSO. 200 μL of culture medium was added to the medium, and then 20 µL of MTT reagent 5 mg/mL MTT in Phosphate Buffer Saline (PBS) was added. The mixture was then left to sit at 37°C for 4 h. After the medium was taken away, 100 µL of DMSO was added. An HT Multi-Detection Microplate Reader from Bio-Tek in Winooski, VT, United States, was used to measure the absorbance at 540 nm and find out what percentage of cells were still alive (Elbouzidi et al., 2023). The study evaluated the effect of the studied extracts on cell viability by measuring absorbance using the following equation:

$$\text{Cell viability }\left(\%\right)=100-\left[\left(\left(A_{0}-A_{t}\right)/A_{0}\right)\times 100\right]$$

A_0_ = absorbance of cells treated with 0.1% DMSO medium, and A_t_ = absorbance of cells treated with the studied extracts at various concentrations. As a negative control, 0.1% DMSO was added to the medium. The IC_50_ values were found using the software GraphPad Prism 8.01 and cisplatin was used as the standard. PBMCs were isolated from human blood samples using Ficollhypaque density centrifugation as per the manufacturer’s instructions (Capricorn Scientific). The studies involving [human] participants were reviewed and approved by the Research Ethics Committee (03/22-LAPABE-10 and 4 March 2022) prior to the experiment. To assess the cytotoxic effects of fig leaf hydro-ethanol extracts on peripheral blood mononuclear cells (PBMCs), the same conditions and concentrations used for tumor cells were utilized.

### 2.8 Molecular docking study

The hydro-ethanol extracts of *F. carica* leaves were tested for their antioxidant, antimicrobial, and anticancer capabilities. The Schrodinger Suite’s Maestro 11.5 software was used to perform the molecular docking analysis. This study set out to discover how the compounds contained in the hydro-ethanol extracts from the *F. carica* leaves interact with the active sites of particular target proteins. These proteins include NADPH oxidase, beta-ketoacyl-[acyl carrier protein] synthase, nucleoside diphosphate kinase, sterol 14-alpha demethylase (CYP51), and caspase-3.

#### 2.8.1 Protein preparation

The crystal structures of the target proteins, namely, NADPH oxidase (PDB ID: 2CDU), beta-ketoacyl-[acyl carrier protein] synthase from *E. coli* (PDB ID: 1FJ4), nucleoside diphosphate kinase from *S. aureus* (PDB ID: 3Q8U), sterol 14-alpha demethylase (CYP51) from *C. albicans* (PDB ID: 5FSA), and caspase-3 were obtained from the RCSB database. The process of protein preparation was carried out using the Protein Preparation Wizard of Maestro 11.5. This involved a series of phases, including preprocessing, refining, and reduction. Hydrogen atoms were introduced, and hydroxyl groups, water molecules, and amino acids were rearranged to correct structural defects such as atom overlap or absence. The proteins were subsequently modified in a delicate manner to enhance their structural characteristics (Kumar et al., 2022; Lafraxo et al., 2022; Tourabi et al., 2023).

#### 2.8.2 Ligand preparation

For ligand generation, the Schrödinger suite’s Ligprep wizard in Maestro 11.5 was used. This requires utilizing the OPLS3 force field to reduce structures, add hydrogen atoms, resolve bond length and angle issues, and transform 2D structures into 3D. The ionization states were fine-tuned while maintaining chirality (Aboul-Soud et al., 2022).

### 2.9 Statistical analysis

Microsoft Office Excel 2021 was utilized in order to determine the correlation coefficients (R2) for the spectrophotometric experiments. Statistical analysis was performed using IBM SPSS Statistics V21.0 software for descriptive statistics, for comparing averages with ANOVA one-way of studied characteristics by *post hoc* (Tukey’s test) at the 5% threshold, for bivariate Pearson correlation analysis with a significance level of P < 0.01 and for the Principal Component Analysis (PCA).

## 3 Results and discussions

### 3.1 Spectrophotometric analysis of bioactive components in hydro-ethanol leaf extracts

Total polyphenols, flavonoids, condensed tannins, and vitamin C were tested in hydro-ethanol extracts from the leaves of the fig plant that was being studied. Table 1 shows that these chemicals were present in the extracts in varying amounts. The synthesis and accumulation of phenolic compounds can be influenced by several internal and external stimuli, such as stress, damage, dryness, and the invasion of pathogens. Some plant species make phenylpropanoid chemicals when they are exposed to nutritional stressors like nitrogen, phosphate, potassium, sulfur, magnesium, boron, and iron, as well as when they are exposed to photo-inhibition. Furthermore, exposure to light stimulates the process of producing phenolic compounds in chloroplasts and storing them in vacuoles (Bhattacharya et al., 2010).

**TABLE 1:** Results of biological compounds analysis in hydro-ethanol leaf extracts from the four Eastern Moroccan fig trees (*Ficus carica* L.).

Extract Reference	Polyphenols (mg GAE/g DW)	Flavonoids (mg QE/g DW)	Condensed tannins (mg CatE/g DW)	Vitamin C (mg Asc A/100 g DW)
CHHEE	58.5 ± 1.6^b^	25.8 ± 0.0^a^	2.4 ± 0.0^d^	8.2 ± 0.1^a^
GDHEE	60.4 ± 1.0^ab^	25.3 ± 0.3^b^	4.7 ± 0.1^c^	7.8 ± 0.0^b^
MAHEE	62.6 ± 1.3^a^	26.2 ± 0.1^a^	4.8 ± 0.0^b^	2.3 ± 0.1^c^
OHHEE	58.1 ± 1.0^b^	24.5 ± 0.1^c^	4.9 ± 0.1^a^	2.3 ± 0.0^c^

MAHEE, Malha hydro-ethanol extract; GDHEE., Ghoudane hydro-ethanol extract; CHHEE, Chetoui hydro-ethanol extract; OHHEE, Onk Hmam hydro-ethanol extract; GAE, Gallic acid equivalent; QE, Quercetin equivalent; CatE, Catechol equivalent; Asc A, Ascorbic acid; DW, Dry weight. Values are expressed as mean ± Standard Error of Measurement “SEM” (n = 3). Ordinary one-way ANOVA using *post hoc* testing (Tukey’s test) at the 5% threshold. Means followed by a different letter in the same column are significantly different (p < 0.05).

The average amount of polyphenols in Malha hydro-ethanol extract (MAHEE) is 62.6 ± 1.3 mg GAE/g DW. This is higher than Ghoudane hydro-ethanol extract (GDHEE), Chetoui hydro-ethanol extract (CHHEE), and Onk Hmam hydro-ethanol extract (OHHEE), which have 58.1 ± 1.0 mg GAE/g DW. When comparing the means of this leaf characteristic, the difference is only noticeable when MAHEE is compared to CHHEE or OHHEE, not when compared the other leaf types. Phenolic compounds are common secondary metabolites found in plants. They not only help plants work, but they are also good for people’s health because they fight free radicals, quench singlet oxygen, and donate a hydrogen atom or electron to other compounds. Figs contain phenolic chemicals. Indeed, red wine and tea, two well-known sources of phenolic chemicals, have lower phenolic levels than figs (Oliveira et al., 2009; Vallejo et al., 2012).

The average amount of flavonoids in MAHEE was 26.2 ± 0.1 mg QE/g DW, followed by CHHEE, GDHEE, and OHHEE at 24.5 ± 0.1 mg QE/g DW. For this trait, there were also significant differences in the average amounts of flavonoids in fig leaves, except between MAHEE and CHHEE. Flavonoids are a distinct class of natural compounds that are the main active substances in many medicinal plants. They are used to treat a variety of diseases by inhibiting specific enzymes and hormones, as well as stimulating and reducing free radical activity. Flavonoids’ antioxidant action is attributed to their phenolic groups and ability to form chelates with transitional metals (Trifunschi et al., 2015).

The amounts of condensed tannins show that these molecules were not the main phenolic compounds in the different ethanol leaf extracts. Instead, the amounts in each extract were very different (ANOVA at p < 0.05). CHHEE 2.4 ± 0.0 mg Cat E/g DW was the lowest level, and OHHEE 4.9 ± 0.1 mg Cat E/g DW was the most significant amount. Condensed tannins are accountable for the astringency seen in fruits and beverages. Still, they are fundamentally different from hydrolysable tannins because they do not contain any sugars in their molecular structure and are more like flavonoids (Soares et al., 2020).

CHHEE had the greatest average vitamin C content, measuring 8.2 ± 0.1 mg Asc. A/100 g DW, followed by GDHEE, MAHEE, and OHHEE at 2.3 ± 0.1 mg Asc. A/100 g DW. Additionally, ANOVA analysis at a significance threshold of 5% revealed significant differences in vitamin C content among fig leaves, except for the comparison between MAHEE and OHHEE. Vitamin C, or ascorbic acid, is a vital nutrient that plays a crucial role in significant physiological processes within the human body. It operates as an antioxidant and enhances immune system functionality. It is responsive to variables such as the degree of ripeness, storage conditions, and heating conditions. *Ficus carica* figs are excellent natural sources of vitamin C (Nerdy et al., 2023).

### 3.2 Antioxidant activity of hydro-ethanol leaf extracts

Antioxidant activity is critical for the human body because it protects cells from free radicals, which are produced during many oxygen-dependent processes and are responsible for oxidative destruction (Havsteen, 2002). The antioxidant activity of fig leaf extracts was assessed using four distinct methodologies (Table 2). The analysis of the findings revealed that MAHEE has the most potent antioxidants when compared to other leaf extracts.

**TABLE 2:** Antioxidant and free radical scavenging results from the four studied hydro-ethanol leaf extracts.

Extract/Reference	DPPH scavenging capacity IC50 (mg/mL)	β-Carotene bleaching assay (mg/mL)	ABTS scavenging assay (TE µmol/mL)	Total antioxidant capacity *
CHHEE	1.0 ± 0.2^bc^	4.4 ± 0.3^c^	97.5 ± 5.6^b^	113.0 ± 6.5^c^
GDHEE	0.9 ± 0.2^bc^	4.1 ± 0.1^c^	99.2 ± 2.5^b^	133.0 ± 9.2^b^
MAHEE	0.7 ± 0.2^b^	3.0 ± 0.1^b^	87.1 ± 6.2^b^	179.0 ± 2.1^a^
OHHEE	1.3 ± 0.1^c^	5.5 ± 0.1^d^	112.2 ± 5.6^c^	92.9 ± 9.7^d^
Ascorbic acid (Asc A)	0.3 ± 0.1^a^	-	07.2 ± 1.4^a^	-
Butylated hydroxytoluene (BHT)	-	0.1 ± 0.1^a^	-	-

DPPH: 2,2-diphényl 1-picrylhydrazyle; β-Carotene: Beta-Carotene; ABTS: 2,2'-Azinobis[3-éthyl-2,3-dihydrobenzothiazole-6-sulfonate; *: Total antioxidant capacity (TAC) expressed as µg ascorbic acid equivalents/mg extract; TE: Trolox equivalent; MAHEE, Malha hydro-ethanol extract; GDHEE, Ghoudane hydro-ethanol extract; CHHEE, Chetoui hydro-ethanol extract; OHHEE, Onk Hmam hydro-ethanol extract. Ordinary one-way ANOVA using *post hoc* testing (Tukey’s test) at the 5% threshold. Means followed by a different letter in the same column are significantly different (p < 0.05).

The analysis of variance (ANOVA) shows that there is only a significant difference between the highest mean antioxidant activity equivalent (MAHEE) value of 0.7 ± 0.2 and the lowest mean OHHEE value of 1.3 ± 0.1, which is measured in terms of DPPH IC_50_ in mg Asc. A equivalence per milliliter, with a significance level of P < 0.05. In earlier work, Petruccelli et al. (2018) looked at ten different types of Italian *F. carica* leaves and found that fig leaf extracts had antioxidant activity when tested using the DPPH method. The IC_50_ value of the extracts ranged from 0.48 to 6.68 mg/mL, which is consistent with the values obtained in this study (Petruccelli et al., 2018). The results of the multiple comparisons of averages from the β-carotene bleaching experiment suggest that there is no significant difference observed only between GDHEE and CHHEE. The antioxidant potency of MAHEE is the strongest, measuring at 3.0 ± 0.1 mg BHT equivalents/mL. On the other hand, OHHEE has the weakest potency, measuring at 5.5 ± 0.1 mg BHT equivalents/mL.

We used a significance level of 5% to do an analysis of variance (ANOVA). The ABTS scavenging assay only showed significant differences between OHHEE and MAHEE. MAHEE demonstrated the most potent antioxidant activity, measuring 87.1 ± 6.2 TE µmol/mL, whereas OHHEE exhibited the least activity, measuring 112.2 ± 5.6 TE µmol/mL. Ergül et al. (2019) discovered that the IC_50_ values for ABTS radical scavenging activity in methanol and water extracts of fig leaves were 559.39 µg BHT/mL and 428.51 µg BHT/mL, respectively (Ergül et al., 2019).

The differences in total antioxidant capacity (TAC) between the four fig leaf extracts that were looked at are statistically significant, as shown by an ANOVA test with a p-value of less than 0.05. It was found that MAHEE had the highest phosphomolybdenum reduction, at 179.0 ± 2.1 µg Asc. A equivalents/mg extract. This was followed by GDHEE, CHHEE, and OHHEE, which had a phosphomolybdenum reduction of 92.9 ± 9.7 µg Asc. A equivalents/mg extract. In a previous study, Raoufa et al. (2021) assessed the total antioxidant capacity of a fig leaf extract at room temperature after 24 h using a solution of 70% ethanol. The resulting value was 0.569 mg of vitamin C equivalents per gram of extract (Abdel-Rahman et al., 2021).

The spectrophotometric assessment of antioxidant efficacy is carried out by measuring the ability of the biological sample to scavenge the synthetic-colored radical. There are two types of tests that can be done. The first tests for hydrogen atom transfer, and the second, which is more interesting to us, tests for electron transfer. These include the DPPH (2.2-diphenyl-1-picrylhydrazy), ABTS (2.2′-Azinobis (3-Ethylbenzothiazoline-6-Sulphonic Acid), and FRAP (Ferric Reducing Antioxidant Potential) tests. These chemical assays are commonly used due to their simplicity, cost-effectiveness, easy accessibility, and independence from advanced laboratory equipment (Ulewicz-Magulska and Wesolowski, 2019). We can quickly, easily, and cheaply find out how antioxidant-rich food is by using a free radical called 2,2-Diphenyl-1-picrylhydrazyl (DPPH). It is commonly used to test substances for their ability to either scavenge free radicals or donate hydrogen and to check the antioxidant activity of foods. In recent years, it has been employed to quantify antioxidants in intricate biological systems. The DPPH method is applicable to both solid and liquid samples and is not limited to any specific antioxidant component; rather, it assesses the overall antioxidant capacity of the sample. An assessment of total antioxidant capacity aids in comprehending the functional attributes of foods (Shalaby and Shanab, 2013). The ABTS method offers additional versatility since it may be employed at various pH levels, in contrast to DPPH, which is sensitive to acidic conditions. This makes it advantageous for investigating the influence of pH on the antioxidant activity of different substances. It is also beneficial for quantifying the antioxidant activity of samples extracted using acidic solvents. Furthermore, ABTS is soluble in both aqueous and organic solvents, making it valuable for evaluating the antioxidant activity of materials across various media. Most of the time, it is used in a Phosphate Buffer Saline (PBS) with 150 mM NaCl to simulate a serum ionic potential solution. An additional benefit of the ABTS + method is that samples responded swiftly with ABTS in the phosphate-buffered saline (PBS), achieving a steady state within 30 min (Shalaby and Shanab, 2013). Antioxidant capacity detected by the ABTS assay was significantly higher for fruits, vegetables, and beverages compared to that by the DPPH assay. The high-pigmented and hydrophilic antioxidants were better reflected by the ABTS assay than the DPPH assay. The results suggest that the ABTS assay may be more useful than the DPPH assay for detecting antioxidant capacity in a variety of foods (Floegel et al., 2011).

Despite the numerous antioxidant activities of our hydro-ethanol extracts of the *Ficu*s *carica* leaves, it is necessary to conduct a comparative study of extracts of varying polarity, as well as other antioxidant methods such as iron chelation and ferric reducing power, to the best of our knowledge.

A bivariate Pearson correlation analysis (Table 3) was performed to investigate the association between antioxidants and their effectiveness in eliminating free radicals. The findings demonstrated a notable and positive association between total polyphenol content (TPC) and total flavonoid content (TFC), with a correlation coefficient of 0.70. In addition, strong negative relationships were found between total condensed tannins (TCT) and vitamin C (Vit C) in the fig leaves that were studied, with a correlation coefficient of −0.65. Furthermore, there was a weak positive correlation observed between TPC and TCT, as well as between TFC and vit C, with correlation coefficients of 0.40 and 0.17, respectively. Conversely, there was a weak negative correlation observed between TPC and vit C, as well as between TFC and TCT, with correlation coefficients of −0.24 and −0.36, respectively. The findings suggest that the abundance of polyphenols in fig leaves is in line with flavonoids. However, the rise in vit C causes a lack of condensed tannin. Additionally, they suggest that the distribution of these bioactive substances within the leaves is dependent on the specific variety of fig tree.

**TABLE 3:** Bivariate Pearson correlation analysis results regarding relationship between antioxidants and their effectiveness in scavenging free radicals.

Correlation	DPPH	β-carotene	ABTS	TAC	TPC	TFC	TCT	Vit C
DPPH	1,00							
β-carotene	0,99	1,00						
ABTS	0,97	0,97	1,00					
TAC	−0,97	−0,97	−0,92	1,00				
TPC	−0,93	−0,94	−0,85	0,98	1,00			
TFC	−0,89	−0,89	−0,97	0,80	0,70	1,00		
TCT	−0,05	−0,06	0,14	0,25	0,40	−0,36	1,00	
Vit C	−0,01	0,014	−0,08	−0,19	−0,24	0,17	−0,65	1,00

Bivariate Pearson correlation analysis with a significance level of P < 0.01. DPPH., DPPH, Scavenging Capacity IC_50_; β-carotene., β-Carotene Bleaching Assay; ABTS., ABTS, scavenging assay; TAC., total antioxidant capacity; TPC., total polyphenol content; TFC., total flavonoid content; TCT., total condensed tannins; Vit C., Vitamin C.

The findings demonstrated a significant and positive association between the total polyphenol content (TPC) and the total antioxidant capacity (TAC), with a correlation coefficient of 0.98. Furthermore, there was a significant inverse relationship between TPC with β-carotene (−0.94), DPPH (−0.93), and ABTS (−0.85). Similarly, TFC demonstrates a strong and positive association with TAC (0.80), while showing significant negative correlation coefficients with β-carotene (−0.89), DPPH (−0.89), and ABTS (−0.97). It turns out that the polyphenolic and flavonoid parts in the hydro-ethanol extracts of the fig leaves that were studied are mainly responsible for antioxidants’ ability to get rid of harmful free radicals. In contrast, we observe weak correlations between TCT or vit C and ABTS, β-carotene, DPPH, and TAC. This suggests that TCT’s or vit C’s role in neutralizing free radicals in these extracts is diminished.

**FIGURE 1:**
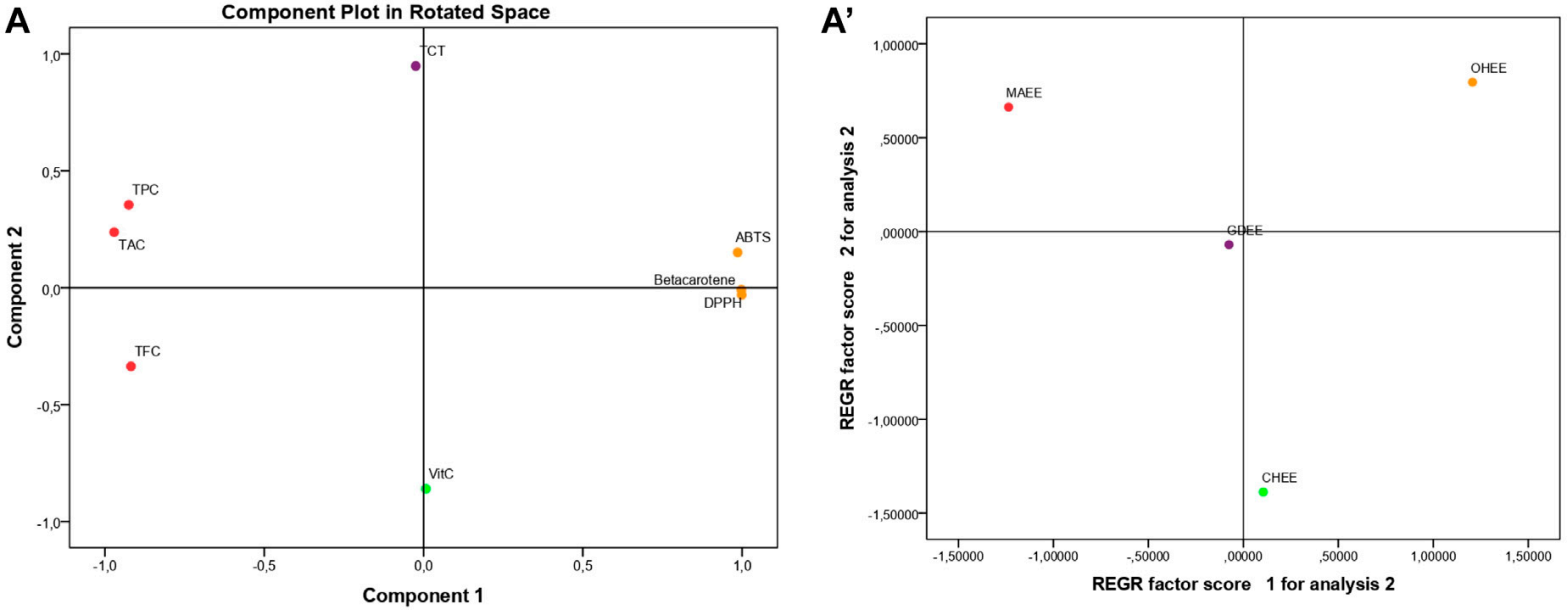
Principal component analysis (PCA); based on the different measurements of bioactive compounds and antioxidant activities in the hydro-ethanol leaf extracts of the four studied fig varieties TPC., Total polyphenol content; TFC., Total flavonoid content; TCT., Total condensed tannins; Vit C., Vitamin C; DPPH., DPPH Scavenging Capacity IC_50_; Beta carotene., β-Carotene Bleaching Assay; ABTS., ABTS Scavenging Assay; TAC., Total Antioxidant Capacity; MAHEE., Malha hydro-ethanol extract; GDHEE., Ghoudane hydro-ethanol extract; CHHEE., Chetoui hydro-ethanol extract; OHHEE., Onk Hmam hydro-ethanol extract.

Principal Component Analysis (PCA), as seen in Figure 1, accounts for 94.558% of the overall variance. Specifically, Component 1 explains 70.110% of the variance, while Component 2 explains 24.448%. Component 1 exhibits a very high positive correlation with β-carotene (0.999), DPPH (0.997), and ABTS (0.978). It also shows a substantial negative correlation with TAC (−0.981), TPC (−0.941), and finally TFC (−0.902). Component 2 exhibits a very significant positive correlation with TCT (0.945). Conversely, it shows a negative correlation with vitamin C (−0.859).

The regression (Figure 1) shows that the leaf extracts are spread out, which means that their bioactive components and antioxidant activity are very different from one another. MAHEE is notable for its high total antioxidant capacity (TAC), polyphenol content (TPC), and flavonoid content (TFC). These outstanding results can be attributed to the variety factor and/or the insufficient maintenance of the trees (due to the salty flavor, known as Malha in Arabic, of the figs they produce and their low market value), so they are vulnerable to stress. As a result of stress, the leaves produce a high concentration of polyphenols and flavonoids, increasing their resistance to cell and tissue damage as well as their antioxidant activity. CHHEE is distinguishable from the other leaf extracts studied by its high vitamin C content and low total condensed tannin content. In comparison to MAHEE, OHHEE has the lowest antioxidant strength (highest ABTS, β-carotene, and DPPH IC_50_). However, unlike CHHEE, it is rich in total condensed tannins and low in vitamin C. GDHEE’s pivotal location shows that the mix of its bioactive chemicals is unique. Its composition is more like any extract than the others analyzed. The findings amply illustrated the effect of the fig variety on the bioactive compounds and the antioxidant activities of their leaves.

### 3.3 HPLC-UV phytochemical analysis

Table 4 shows that eight different phytochemicals, such as polyphenols and flavonoids, were found in the fourth hydro-ethanol leaf extract that was studied. The main parts of all the tested extracts were found to be caffeic acid, rutin, and trans ferulic. The active compounds cathechine for MAHEE, naringin for CHHEE, and kaempferol for GDHEE were only found in one of the fig leaf extracts. Myricitin is only present in CHHEE and OHHEE, whereas quercetin is completely absent in GDHEE.

**TABLE 4:** HPLC- UV phenolic profile of ethanol extracts from the studied fig leaves.

Compounds	Chemical formula	Structural formula	Classification	RT (min)	Area %			
					CH HEE	GD HEE	MA HEE	OH HEE
Catechin	C_15_H_14_O_6_	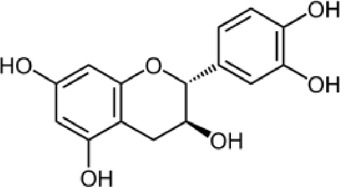	Flavonoid flavonols	12.0	ND	ND	2.20	ND
Caffeic acid	C_9_H_8_O_4_	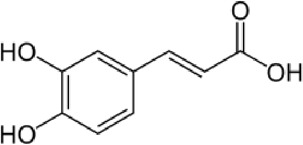	Hydroxy cinnamic acid	13.9	38.79	40.58	33.34	32.92
Trans ferulic	C_10_H_10_O_4_	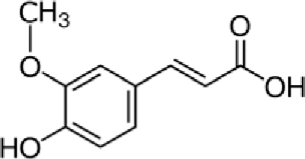	Hydroxy cinnamic acid	14.8	12.86	9.35	13.21	7.61
Rutin	C_27_H_30_O_16_	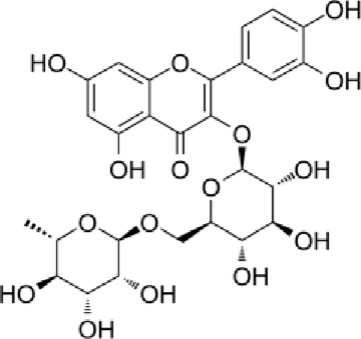	Flavonoid glycosides	18.4	24.37	21.82	31.04	16.74
Naringin	C_27_H_32_O_14_	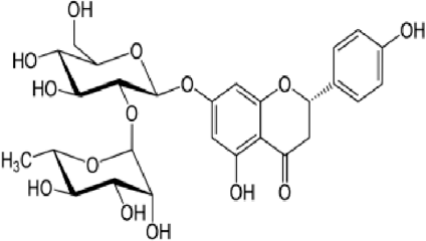	Flavanone glycosides	20.2	1.11	ND	ND	ND
Myricitin	C_15_H_10_O_8_	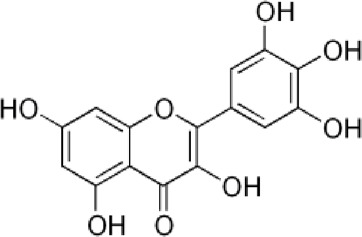	Flavonoid flavonols	25.2	0.29	ND	ND	1.16
Kaempferol	C_15_H_10_O_6_	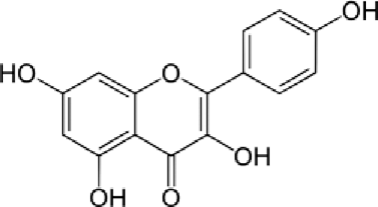	Flavonoid flavonols	42.2	ND	3.37	ND	ND
Quercetin	C_15_H_10_O_7_	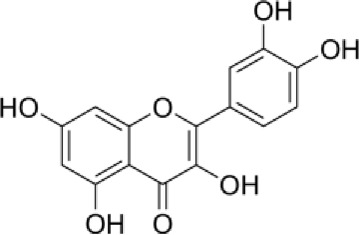	Flavonoid flavonols	44.5	02.93	ND	1.47	3.55

RT., retention time; ND., Not detected; MAHEE., Malha hydro-ethanol extract; GDHEE., Ghoudane hydro-ethanol extract; CHHEE., Chetoui hydro-ethanol extract; OHHEE., Onk Hmam hydro-ethanol extract.

Caffeic acid, a phenolic compound, is produced by plants and can be found in beverages including coffee, wine, tea, and propolis (Alam et al., 2022; Mirzaei et al., 2021). Furthermore, this phenolic acid and its derivatives possess antioxidant properties, in addition to their anti-carcinogenic and anti-inflammatory qualities. It has been shown to help fight hepatocarcinoma (HCC) in both *in vitro* and *in vivo* studies (Espíndola et al., 2019). It also contributes to the plant’s defense against diseases and other destructive organisms by suppressing the growth of bacteria, fungus, and insects (Khan et al., 2021; Ramaroson et al., 2022; Riaz et al., 2019).

Rutin is the main compound of flavonoid glycosides found in Ficus (Vaya and Mahmood, 2006). It is possible to hydrolyze one terminal rhamnose into quercetin. Alternatively, it can be converted into isoquercitrin, which has higher bioavailability and stronger anti-proliferative properties compared to rutin and quercetin (Takahama et al., 2009). Rutin, an essential dietary component, influences the fragility and permeability of capillaries, hence enhancing human health (Kamalakkannan and Prince, 2006). It has various medical uses, such as being an antioxidant, an antiallergen, an antibacterial, an antiulcer, an anticarcinogenic, an anti-inflammatory, an anti-diabetic, and an antimutagenic substance (Caglayan et al., 2019; Li et al., 2010). When given orally to rats, its antioxidant activity and bioavailability were shown to be lower than those of quercetin. In humans, quercetin’s bioavailability in capsule form is less than 1%, mainly due to its low absorption in the small intestine (Erlund, 2004).

Ferulic acid, a widely distributed phenolic acid found in plants, is a constituent of Chinese medicinal herbs. Usually, it combines with mono- and oligosaccharides, polyamines, lipids, and polysaccharides in plants and it demonstrates low toxicity and can be readily absorbed and digested. Scientific evidence has demonstrated that ferulic acid possesses antioxidant, antimicrobial, anti-inflammatory, anti-thrombotic, and anti-cancer properties. Furthermore, it offers defense against heart disease, lowers cholesterol levels, and improves sperm viability. It is extensively utilized in the food and cosmetic sectors due to its desirable qualities and minimal toxicity. It is the main component that is used in the production of vanillin and preservatives. Moreover, it serves as a cross-linking agent for the production of food gels and edible films. It is also used into sports foods and skin protection products. Ferulic acid can be chemically manufactured or created via biological transformation (Ou and Kwok, 2004).

### 3.4 Antimicrobial activity

The hydro-ethanol extracts derived from the leaves of the four fig varieties exhibited varying degrees of efficacy in eliminating the tested strains (Table 5). The reference medications exhibited the most potent inhibitory effect when compared to the four fig leaf extracts that were examined. This was demonstrated by an ANOVA study, utilizing Tukey’s *post hoc* test, at a significance level of 5%. The increased antimicrobial efficacy of MAHEE and GDHEE, in comparison to CHHEE and OHHEE, can be attributed to their elevated levels of bioactive constituents, such as polyphenols and flavonoids.

**TABLE 5:** Disk diffusion assay results for the investigated leaf extracts and reference drugs.

Fig leaf extracts		Microbial strains tested				
		*E. coli*	*P. aeruginosa*	*S. aureus*	*B. subtilis*	*C. albicans*
CHHEE		12.6 ± 0.1^b^	11.8 ± 0.6^c^	10.5 ± 0.3^c^	12.6 ± 0.1^c^	18.65 ± 0.2^c^
GDHEE		12.85 ± 0.2^b^	13.9 ± 1.0^b^	11.5 ± 0.1^b^	12.8 ± 0.2^c^	18.9 ± 0.1^c^
MAHEE		12.9 ± 0.1^b^	13.95 ± 0.8^b^	11.6 ± 0.2^b^	14.0 ± 0.3^b^	20.4 ± 0.1^b^
OHHEE		12.5 ± 0.4^b^	11.7 ± 0.4^c^	10.4 ± 0.2^c^	12.5 ± 0.4^c^	18.6 ± 0.2^c^
Controls	Gentamicin	28.5 ± 0.1^a^	23.5 ± 0.4^a^	26.4 ± 0.2^a^	20.5 ± 0.2^a^	N.T
	Fluconazole	N.T	N.T	N.T	N.T	25.8 ± 0.2^a^
	DMSO	0	0	0	0	0

N.T, Not Tested; 0, No Activity; *E. coli*, *Escherichia coli* ATCC 25922; *P. aeruginosa*, *Pseudomonas aeruginosa* ATCC 27853; *S. aureus*, *Staphylococcus aureus* ATCC 25923; *B. subtilis*, *Bacillus subtilis* subsp. Spizizenii ATCC 6633; *C. albicans*, *Candida albicans* (Clinical isolated); MAHEE, Malha hydro-ethanol extract; GDHEE, Ghoudane hydro-ethanol extract; CHHEE, Chetoui hydro-ethanol extract; OHHEE, Onk Hmam hydro-ethanol extract; DMSO, Diméthyl Sulfoxide. Inhibition zones including the diameter of the paper disc (6 mm). Each value is represented as mean standard deviation, (n = 3), Means followed by a different letter in the same column are significantly different (ANOVA - by *post hoc* Tukey’s test p < 0.05).

It is noteworthy to highlight that the hydro-ethanol extracts from the examined fig leaves were quite effective against *C. albicans*. The ANOVA analysis revealed that the differences were only significant between MAHEE and the other leaf hydro-ethanol extracts. The OHHEE had the lowest effect (18.6 ± 0.2 mm), followed by CHHEE (18.65 ± 0.2 mm) and GDHEE (18.9 ± 0.2 mm), while MAHEE had the strongest effect (20.4 ± 0.1 mm). For gram-negative bacteria, there is no significant difference in inhibitory activity against Escherichia *coli* among the leaf extracts. MAHEE had the highest effect (12.9 ± 0.1 mm), followed by GDHEE (12.85 ± 0.2 mm) and CHHEE (12.6 ± 0.1 mm), while OHHEE had the lowest effect (12.5 ± 0.4 mm). MAHEE (13.95 ± 0.8 mm) and GDHEE (13.9 ± 1.0 mm) had the highest activity against *P. aeruginosa*, while CHHEE (11.8 ± 0.6 mm) and OHHEE (11.7 ± 0.4 mm) had smaller inhibition diameters. However, there was no significant difference in inhibitory activity between MAHEE and GDHEE or between CHHEE and OHHEE. In relation to Gram-positive bacteria, the OHHEE (10.4 ± 0.2 mm) and CHHEE (10.5 ± 0.3 mm) exhibited the least efficacy against *S. aureus*. Conversely, the GDHEE (11.5 ± 0.1 mm) and MAHEE (11.6 ± 0.2 mm) demonstrated the greatest diameters of inhibition. The inhibitory actions of OHHEE and CHHEE, as well as GDHEE and MAHEE, did not differ significantly. In addition, it was observed that leaf extracts exhibited inhibitory effects on *B. subtilis*. Among these, MAHEE demonstrated the most pronounced inhibitory effect (14.0 ± 0.3 mm), followed by GDHEE (12.85 ± 0.2 mm) and CHHEE (12.6 ± 0.1 mm). OHHEE exhibited the least effective effect (12.5 ± 0.4 mm). Furthermore, no statistically significant distinction in inhibitory action was found between GDHEE, CHHEE and OHHEE.

Resistance and sensitivity are not invariably associated with a singular substance or molecular class; they are intricate systems that involve multiple types of molecules (Coulibaly et al., 2020). The concentration and composition of bioactive chemicals and minerals may have influenced the extracts’ antimicrobial effect against different strains. Certain minerals and bioactive compounds, such as polyphenols, can interact to enhance or reduce antimicrobial activity in plants and extracts, respectively (Cunningham et al., 2010). Many recent investigations have shown that medicinal mineral compounds serve as antimicrobials by interacting synergistically with bioactive components found in plant extracts such as polyphenols, flavonoids, terpenoids, and others, or by altering pH, These interactions can either augment or diminish the antimicrobial characteristics of the plant or the antibacterial efficacy of the extract, contingent upon the specific chemical elements present in minerals and bioactive chemicals (Divakar et al., 2018). Plants produce flavonoids and coumarins as a protective mechanism against microbial infections. The ability of these compounds to interact with extracellular and soluble proteins, as well as bacterial cell walls, may contribute to their claimed efficacy. Furthermore, lipophilic flavonoids have the ability to damage microbial membranes and act as bioactive molecules that interact with microorganisms in a variety of ways, impacting their development and survival (Tsuchiya et al., 1996). Several studies have found that *F. carica* leaf extracts exhibit antibacterial properties against oral bacteria, nosocomial infectious agents, food poisoning bacteria, fungi, and viruses (Jeong et al., 2009; Wang et al., 2004).

The minimum inhibitory concentrations (MIC) of the leaf extracts were analyzed (Table 6). All of the ethanol extracts from the varieties that were studied had the same MIC values for the two strains that were tested: 64 μg/mL against *E. coli* and 32 μg/mL against *C. albicans*. It also demonstrated that the ethanol extracts from both MA and GD varieties, as well as those from CH and OH, had equal MIC values against the other strains examined. However, MAHEE and GDHEE showed a MIC of 32 μg/mL against *P. aeruginosa*, whereas CHHEE and OHHEE showed a MIC of 64 μg/mL. The MAHEE and GDHEE showed a MIC of 128 μg/mL against *S. aureus*, while the CHHEE and OHHEE had a MIC of 256 μg/mL. *Bacillus subtilis* showed a higher sensitivity to MAHEE and GDHEE, with a MIC of 128 μg/mL. In contrast, it demonstrated lesser sensitivity to CHHEE and OHHEE, with a MIC of 256 μg/mL.

**TABLE 6:** Minimum inhibitory concentrations (MIC values) in µg/mL for the investigated leaf extracts.

Fig leaf extracts	*E. coli*	*P. aeruginosa*	*S. aureus*	*B. subtilis*	*C. albicans*
CHHEE	64 ± 0.0^a^	64 ± 0.0^b^	256 ± 0.0^b^	256 ± 0.0^b^	32 ± 0.0^a^
GDHEE	64 ± 0.0^a^	32 ± 0.0^a^	128 ± 0.0^a^	128 ± 0.0^a^	32 ± 0.0^a^
MAHEE	64 ± 0.0^a^	32 ± 0.0^a^	128 ± 0.0^a^	128 ± 0.0^a^	32 ± 0.0^a^
OHHEE	64 ± 0.0^a^	64 ± 0.0^b^	256 ± 0.0^b^	256 ± 0.0^b^	32 ± 0.0^a^

*E. coli*, *Escherichia coli* ATCC 25922; *P. aeruginosa*, *Pseudomonas aeruginosa* ATCC 27853; *S. aureus*, *Staphylococcus aureus* ATCC 25923; *B. subtilis*, *Bacillus subtilis* subsp. Spizizenii ATCC 6633; *C. albicans*, *Candida albicans* (Clinical isolated); MAHEE, Malha hydro-ethanol extract; GDHEE, Ghoudane hydro-ethanol extract; CHHEE, Chetoui hydro-ethanol extract; OHHEE, Onk Hmam hydro-ethanol extract. Each MIC value is represented as mean standard deviation, (n = 3), Means followed by a different letter in the same column are significantly different (ANOVA - by *post-hoc* Tukey’s test p < 0.05).

The minimum inhibitory concentration (MIC) is determined by several factors, including the botanical source and specific plant component used for extraction, the extraction process (including temperature and duration), the solvent used, the amount of active components extracted, and the specific microorganism that the extract is intended to inhibit (Tikent et al., 2023).

### 3.5 Anticancer activity against breast cancer cell lines

Cancer is one of the world’s most serious health problems and the second-biggest cause of mortality after cardiovascular disease (Tayoub et al., 2018). Conventional clinic therapies such as chemotherapy, surgery, and radiotherapy have a number of dangerous side effects and can damage noncancerous tissues (Birjandian et al., 2018). Furthermore, with rising medication resistance, particularly in cancer treatment, plants have become increasingly significant in the quest for novel chemotherapeutic agents. Many anticancer medicines originating from plants are used in clinics, including vincristine, vinblastine (*Catharanthus* sp.), paclitaxel (*Taxus* sp.), and epipodophyllotoxins (*Podophyllum* sp.) (Sengupta et al., 2017).

**FIGURE 2:**
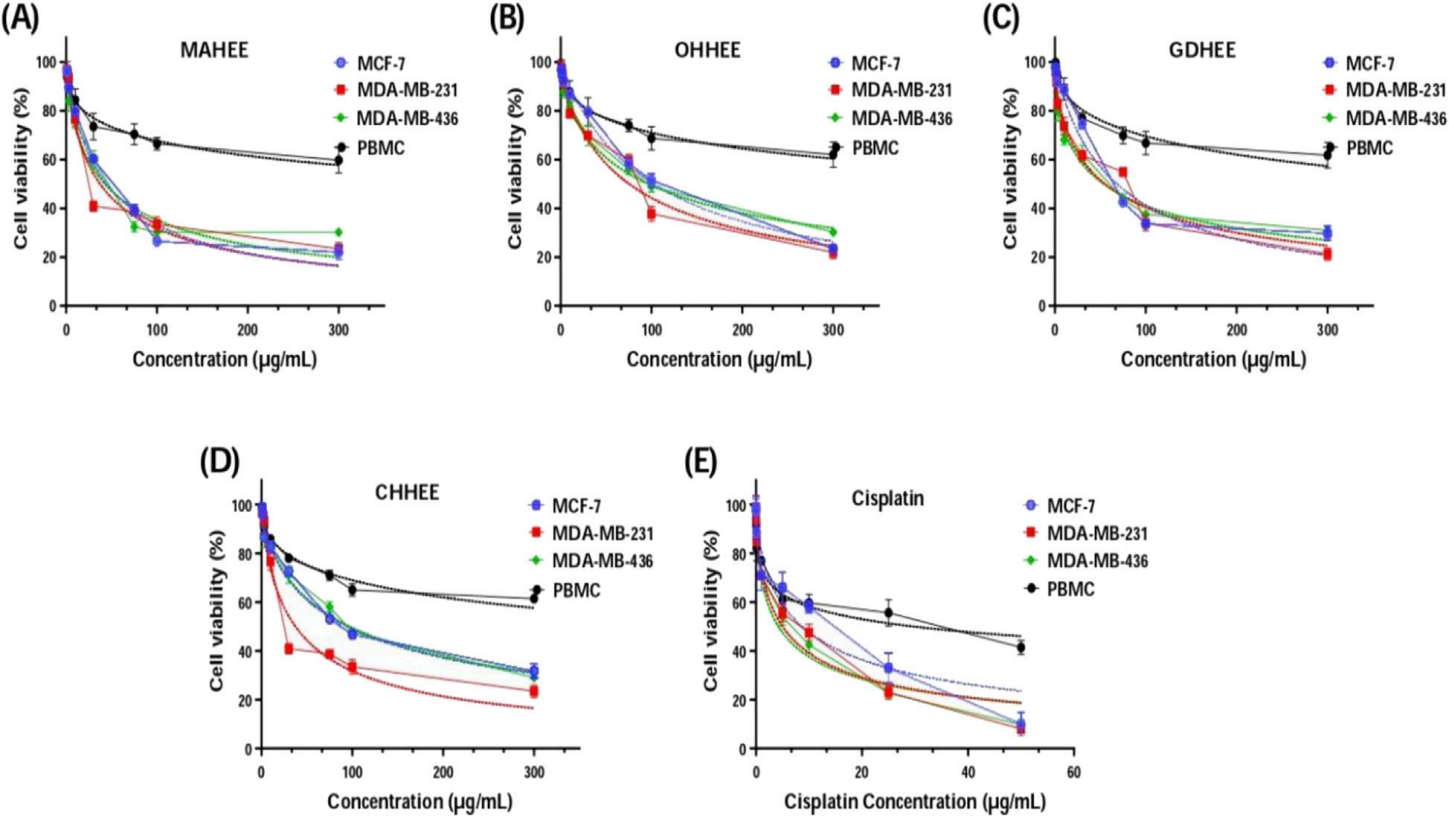
Cell viability of MCF-7, MDA-MB-231, MDA-MB-436 and PBMC cells after 72 h of treatment with F*. carica* hydro-ethanol extracts: **(A)** MAHEE, **(B)** OHHEE, **(C)** GDHEE, **(D)** CHHEE and **(E)** cisplatin as positive control, using MTT test. MAHEE., Malha hydro-ethanol extract; GDHEE., Ghoudane hydro-ethanol extract; CHHEE., Chetoui hydro-ethanol extract; OHHEE., Onk Hmam hydro-ethanol extract.

Upon analyzing the results presented in Figure 2 and Table 7, it is evident that the IC_50_ values, which indicate the concentration needed to inhibit 50% of cell growth, reveal that the most potent and distinguishable extracts are MAHEE and GDHEE for MCF-7 and MDA-MB-436. Similarly, for MDA-MB-231, the extracts MAHEE and CHHEE exhibit the strongest effects. The IC_50_ values of these extracts are the closest to the lowest IC_50_ values of cisplatin, the conventional chemotherapeutic medication. In terms of selectivity index, cisplatin demonstrates the least selectivity when compared to all ethanol extracts of fig leaves that were tested. On the other hand, MAHEE exhibits the highest selectivity index among the three cancer cell lines examined, with values of 14.91 for MCF-7, 17.79 for MDA-MB-231, and 15.57 for MDA-MB-436. Polyphenols can halt or even reverse cancer progression by interacting with molecules in the intracellular signaling network that are involved in cancer initiation and promotion. By influencing a small number of critical components of cellular signaling, polyphenols can also induce apoptosis in cancer cells (Link et al., 2010).

**TABLE 7:** IC_50_ and selectivity indexes of leaf hydro-ethanol extracts on the examined cancer cell lines.

	IC_50_ value ± SD (µg/mL) *				Selectivity index**		
	MCF 7	MDA-MB 231	MDA-MB 436	PBMC	MCF 7	MDA-MB 231	MDA-MB 436
MAHEE	44.5 ± 4.3^b^	37.3 ± 1.4^b^	42.6 ± 2.3^b^	663.6 ± 11.2^b^	14.91	17.79	15.57
OHHEE	102.4 ± 2.2^d^	75.4 ± 3.2^d^	96.2 ± 5.5^d^	791.4 ± 5.8^a^	7.72	10.49	8.22
GDHEE	68.1 ± 5.4^c^	55.1 ± 1.9^c^	53.6 ± 1.8^c^	550.5 ± 9.1^d^	8.08	9.99	10.27
CHHEE	93.9 ± 7.3^d^	39.5 ± 2.6^b^	96.9 ± 4.7^d^	594.5 ± 4.1^c^	6.33	15.05	6.13
Cisplatin	8.6 ± 1.3^a^	5.0 ± 0.1^a^	4.2 ± 0.6^a^	29.6 ± 2.1^e^	3.44	5.92	7.04

IC, Inhibitory Concentration; MCF7, Michigan Cancer Foundation-7; MDA-MB 231, Monroe Dunaway Anderson -Metastasic Breast 231; MDA-MB 436, Monroe Dunaway Anderson -Metastasic Breast 436; PBMC, Peripheral Blood Mononuclear Cell MAHEE, Malha hydro-ethanol extract; GDHEE, Ghoudane hydro-ethanol extract; CHHEE, Chetoui hydro-ethanol extract; OHHEE, Onk Hmam hydro-ethanol extract. * Values are obtained from three independent experiments and expressed as means ± SD (standard deviation). ** Selectivity index = (IC_50_ of PBMC/IC_50_ of tumor cells), PBMCs are the first normal cell populations to come into contact with anticancer medicines employed in the conventional intravenous chemotherapy of patients.

To sum up, the data strongly support the good selectivity profile of the hydro-ethanol fig leaf extracts, especially MAHEE and GDHEE for MCF-7 and MDA-MB-436 and MAHEE and CHHEE for MDA-MB-231. The results indicate that fig leaf extracts possess promising anti-cancer effects, necessitating additional research, such as mechanistic studies and *in vivo* trials, to authenticate their efficacy. Further research into the potential application of these extracts in cancer therapy, combined with an in-depth understanding of their mechanisms of action, will significantly enhance their clinical applicability.

### 3.6 *In silico* analysis of the antioxidant, antimicrobial, and anticancer activity of the components found in the hydro-ethanol extract of leaves

NADPH oxidase (NOX) is a crucial enzyme involved in the generation of reactive oxygen species (ROS), and its function is closely regulated within cells. NADPH oxidase-generated reactive oxygen species (ROS) play a crucial role in protecting against pathogens and facilitating cellular communication. Many people think that oxidative stress, which is caused by NADPH oxidase making too many reactive oxygen species (ROS), is the main reason why tissues get damaged in a number of respiratory inflammatory diseases and injuries, such as asthma, cystic fibrosis, acute respiratory distress syndrome (ARDS), and chronic obstructive pulmonary disease (COPD). Suppressing the activity of NADPH oxidase is a highly effective method to enhance the antioxidant activity and provide protection against diseases associated with oxidative stress (Lee and Yang, 2012). NADPH oxidase inhibitors have the potential to preserve cellular wellbeing and restrain disease progression by reducing reactive oxygen species (ROS) generation, enhancing cellular antioxidant mechanisms, and mitigating chronic inflammation.

**FIGURE 3:**
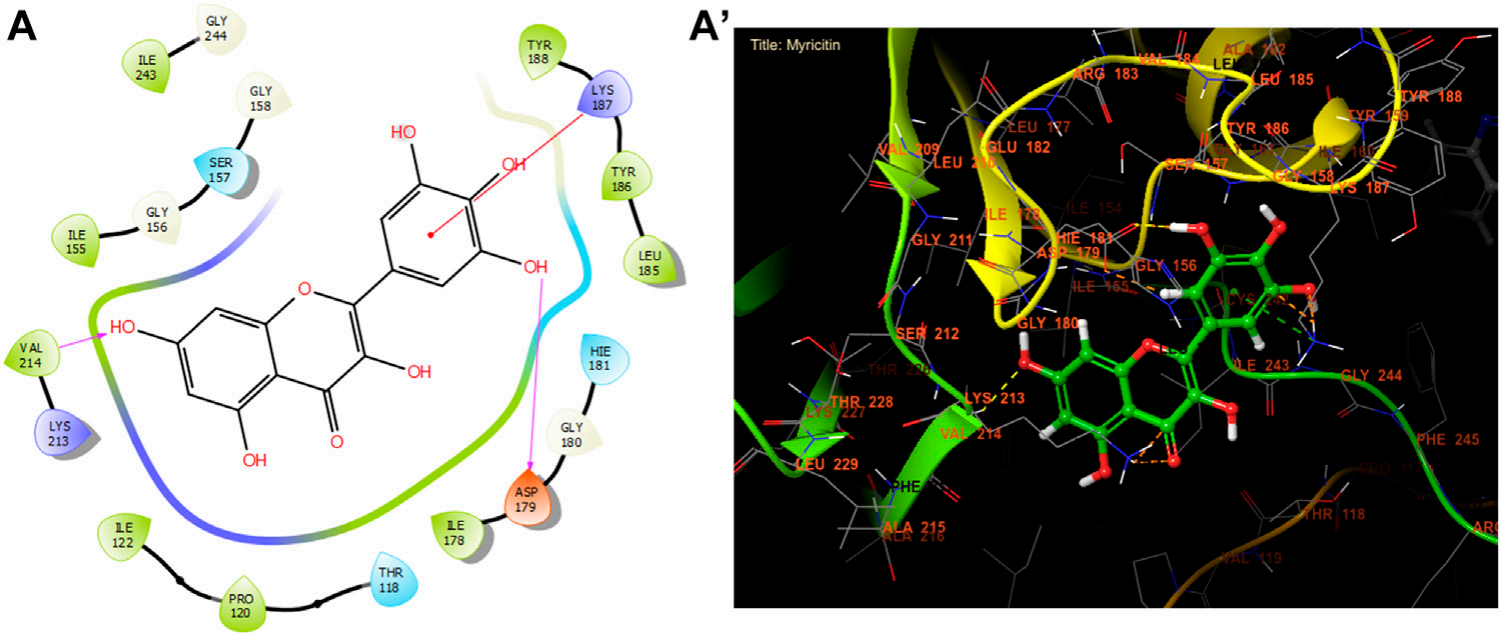
The ligand “Myricitin” interacts with the active site of NADPH oxidase through the 2D viewer **(A)** and the 3D viewer **(A’)**.

According to Figure 3 and Table 8, our *in silico* research revealed that the three most active compounds from leaf extracts against NADPH oxidase were myricitin, quercetin, and rutin. These compounds had glide gscores of −6.59, −6.58, and −5.97 kcal/mol, respectively. Myricitin has formed two hydrogen bonds with VAL 214 and ASP 179, and a single Pi cation bond with LYS 187 in the active region of NADPH oxidase.

**TABLE 8:** Glide gscore based on targeted antioxidant protein and bioactive compounds of leaf extracts.

NADPH oxidase (PDB ID: 2CDU)	Leaf extracts bioactive compounds							
	Caffeic acid	Catechin	Kaempferol	Myricitin	Naringin	Rutin	Quercetin	Trans ferulic
Glide gscore (Kcal/mol)	−5.48	−5.55	−5.54	−6.59	−5.16	−5.97	−6.58	−5.40

Beta-ketoacyl-ACP synthase primarily functions to synthesize fatty acids of different lengths to be utilized by the organism. These applications encompass energy storage and the formation of cell membranes. Fatty acids have the ability to generate various compounds such as prostaglandins, phospholipids, and vitamins, among other substances (Witkowski et al., 2002). Beta-ketoacyl-ACP synthases are very important for making lipoproteins, phospholipids, and lipopolysaccharides. Because they do so many important things in the body, they have become a focus for the development of antibacterial drugs. Bacteria modify the composition of their membranes by changing the phospholipids in order to adjust to their surroundings. Therefore, blocking this channel could serve as a strategic location for impeding the proliferation of bacteria (Zhang and Rock, 2008).

**FIGURE 4:**
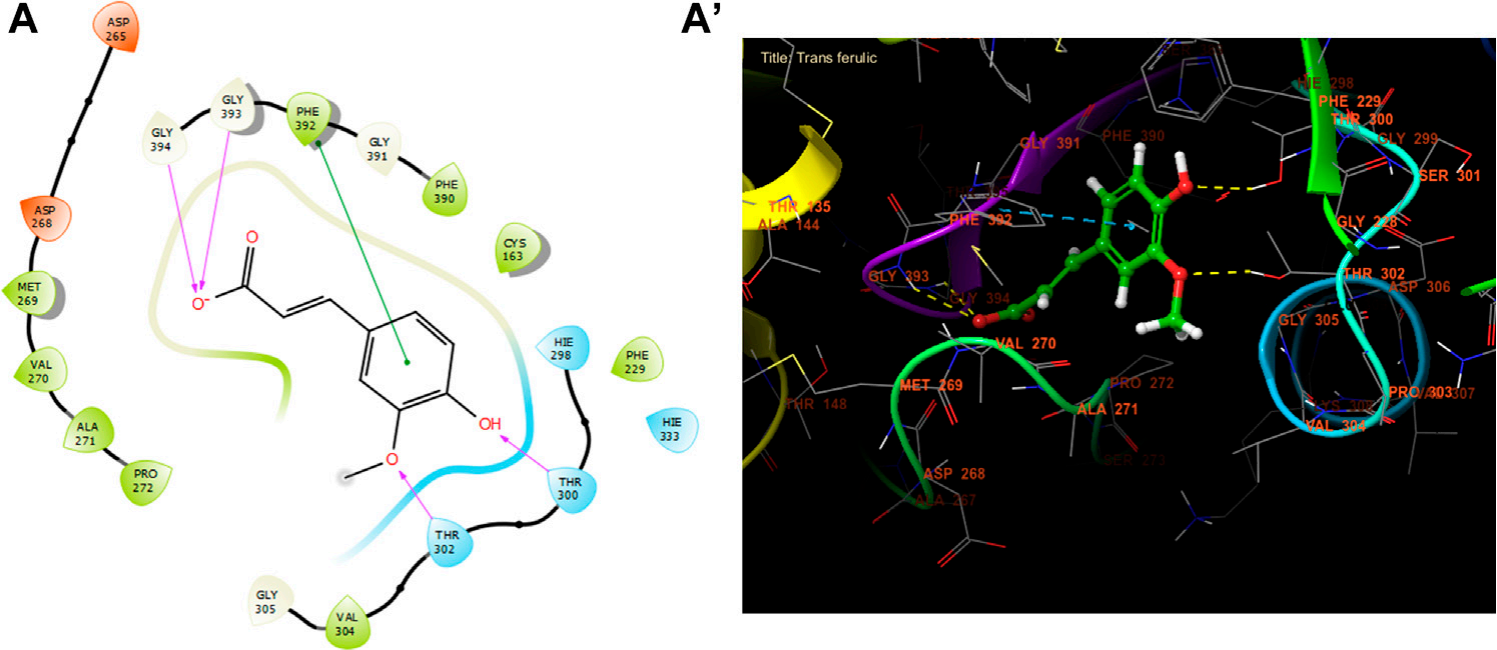
The ligand “Trans ferulic” interacts with the active site of beta-ketoacyl-[acyl carrier protein] synthase through the 2D viewer **(A)** and the 3D viewer **(A’)**.

Trans ferulic, catechin, and caffeic acid had the highest antibacterial efficacy against beta-ketoacyl-[acyl carrier protein] synthase from *E. coli*, with glide gscores of −6.55, −6.45, and −6.42 kcal/mol, respectively (Table 9). Trans ferulic formed four hydrogen bonds with GLY 394, GLY 393, THR 302, and THR 300, and a solitary Pi-Pi stacking bond with PHE 392 in the active region of beta-ketoacyl-[acyl carrier protein] synthase from *E. coli* (Figure 4).

**TABLE 9:** Glide gscore based on targeted microbial proteins and bioactive compounds of leaf extracts.

	Leaf extracts bioactive compounds							
Microbial protein targeted	Caffeic acid	Catechin	Kaempferol	Myricitin	Naringin	Rutin	Quercetin	Trans ferulic
Beta-ketoacyl-[acyl carrier protein] synthase (PDB ID: 1FJ4)	−6.42	−6.45	−5.95	−6.02	***	−4.83	−5.92	−6.55
Nucleoside diphosphate kinase (PDB ID: 3Q8U)	−7.93	−6.79	−8.98	−8.88	−4.67	−7.87	−8.99	−7.93
Sterol 14-alpha demethylase (CYP51) (PDB ID: 5FSA)	−5.39	−7.63	−7.84	−7.84	−5.57	−6.24	−7.62	−5.21

***, No interaction. Glide gscore mesure the interaction between the boactive compound and the protein targeted in (Kcal/mol).

Bacterial diphosphate kinase (Ndk) controls the amount of nucleoside triphosphate (NTP) in cells by moving γ-phosphate from NTPs to NDPs and back again. In addition to its primary function in nucleotide metabolism, Ndk is involved in protein histidine phosphorylation, DNA cleavage/repair, and gene regulation. Ndk also controls bacterial adaptive responses. Ndks made by bacteria inside cells stop many of the host’s defense systems from working. These include phagocytosis, cell apoptosis/necrosis, ROS production, and inflammation. In contrast, extracellular bacteria-secreted Ndks exacerbate host cell cytotoxicity and the inflammatory response. Although Ndks from intra- and extracellular bacteria govern host cellular events in different ways, their basic function is the same, creating the host milieu conducive to colonization and dissemination (Yu et al., 2017). As a result, inhibiting Ndk is an effective strategy for preventing the emergence of bacteria.

**FIGURE 5:**
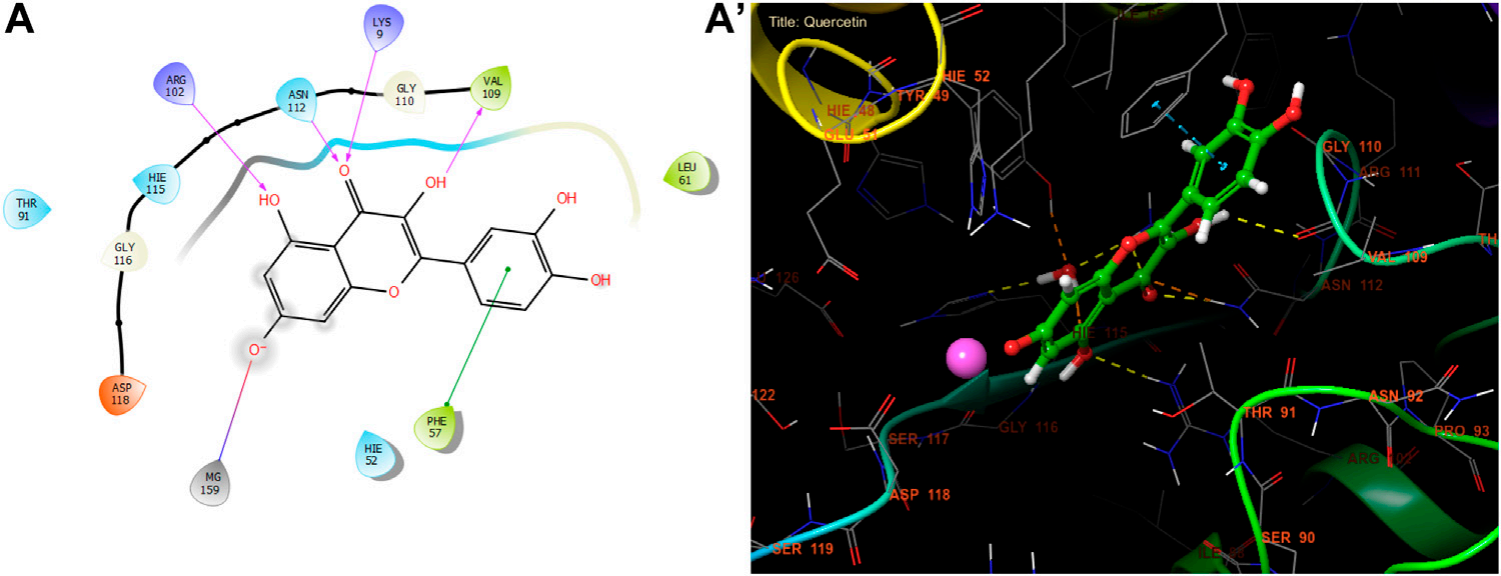
The ligand “Quercetin” interacts with the active site of nucleoside diphosphate kinase through the 2D viewer **(A)** and the 3D viewer **(A’)**.

Quercetin, kaempferol, and myricitin exhibited the highest level of activity against *S. aureus* nucleoside diphosphate kinase, with glide gscore values of −8.99, −8.98, and −8.88 kcal/mol respectively (Table 9). Quercetin formed four hydrogen bonds with residue LYS 9, ASN 112, ARG 102, and VAL 109, and a salt bridge with residue MG 159 at the active site of *S. aureus* nucleoside diphosphate kinase. Additionally, it established a Pi-Pi stacking bond with residue PHE, as seen in Figure 5.

The lanosterol or sterol 14α-demethylase is an enzyme belonging to the cytochrome P450 (CYP) superfamily. It is linked to the CYP51 family, which is a crucial enzyme involved in sterol production in eukaryotes. This enzyme is also a primary target for antifungal azoles and has potential for antiprotozoan chemotherapy. While 14α-demethylase can be found in many different organisms, it is mainly investigated in fungi due to its crucial function in regulating membrane permeability (Daum et al., 1998). In fungi, the enzyme CYP51 helps break down lanosterol, which makes a key building block that is then changed into ergosterol (Lepesheva and Waterman, 2007). This steroid then spreads throughout the cell, modifying plasma membrane permeability and stiffness in a similar manner to cholesterol in animals (Becher and Wirsel, 2012). Due to the essential role of ergosterol in fungal membranes, numerous antifungal drugs have been developed to hinder the activity of 14α-demethylase and thus impede the synthesis of this crucial molecule (Becher and Wirsel, 2012).

**FIGURE 6:**
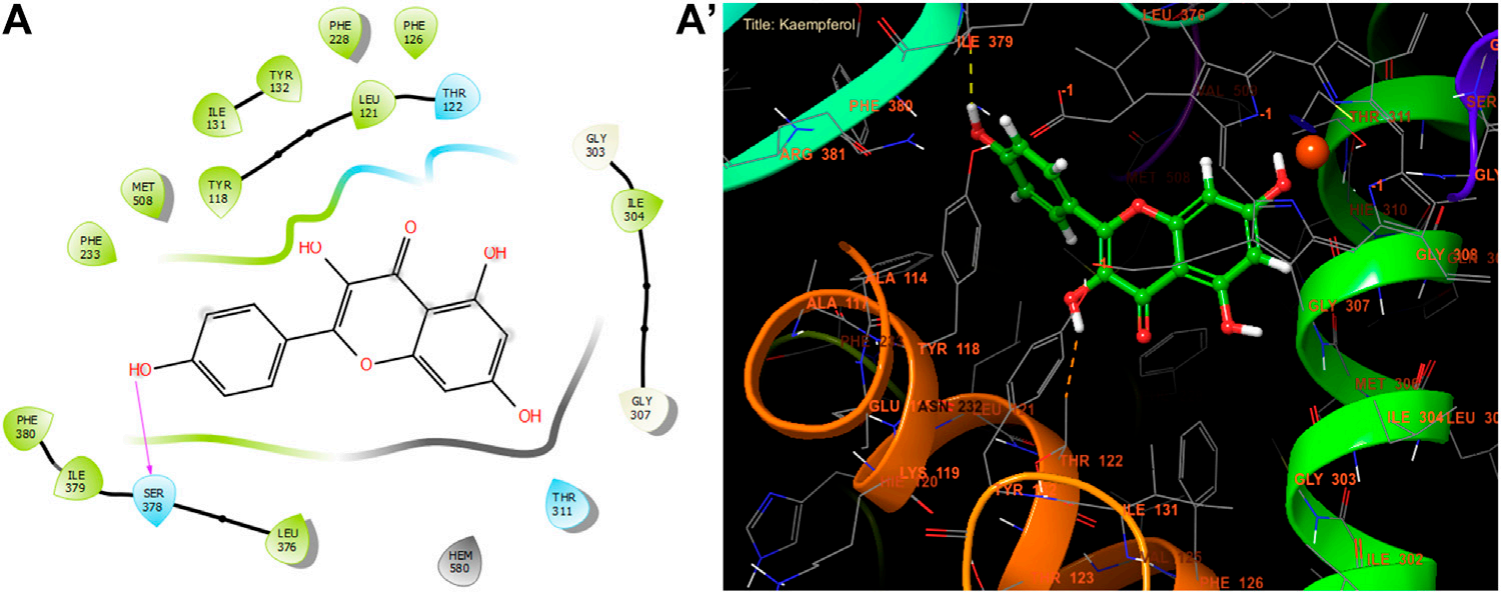
The ligand “Kaempferol” interacts with the active site of sterol 14-alpha demethylase (CYP51). Through the 2D viewer **(A)** and the 3D viewer **(A’)**.

With a glide gscore of −7.84, kaempferol and myricitin had the most powerful effect on sterol 14-alpha demethylase (CYP51) from the harmful fungus *C. albicans*. Catechin came in second, with a glide gscore of −7.63 kcal/mol (Table 9). Kaempferol exhibited antifungal action by forming a singular bond with SER 378 residue in the active region of sterol 14-alpha demethylase (CYP51) derived from the pathogenic *C. albicans* (Figure 6).

Caspase-3 is crucial in the process of fighting cancer by acting as a key player in the pathway of programmed cell death, known as apoptosis, which is necessary for the controlled removal of cancer cells. Caspase-3 is activated by initiator caspases in both the internal and extrinsic pathways of apoptosis. This activation results in the breakdown of essential cellular components and ultimately leads to cell death. Inducing caspase-3 activation can initiate apoptosis in cancer cells, hence counteracting their survival mechanisms and resistance to traditional treatment. In addition, some anticancer strategies focus on enhancing caspase-3 activity using drugs that either increase pro-apoptotic signals or inhibit anti-apoptotic proteins inside the tumor microenvironment. Therefore, utilizing the activation of caspase-3 is a feasible approach in cancer treatment, aiming to induce cell death specifically in cancerous cells while minimizing harm to healthy organs (Asadi et al., 2022).

**FIGURE 7:**
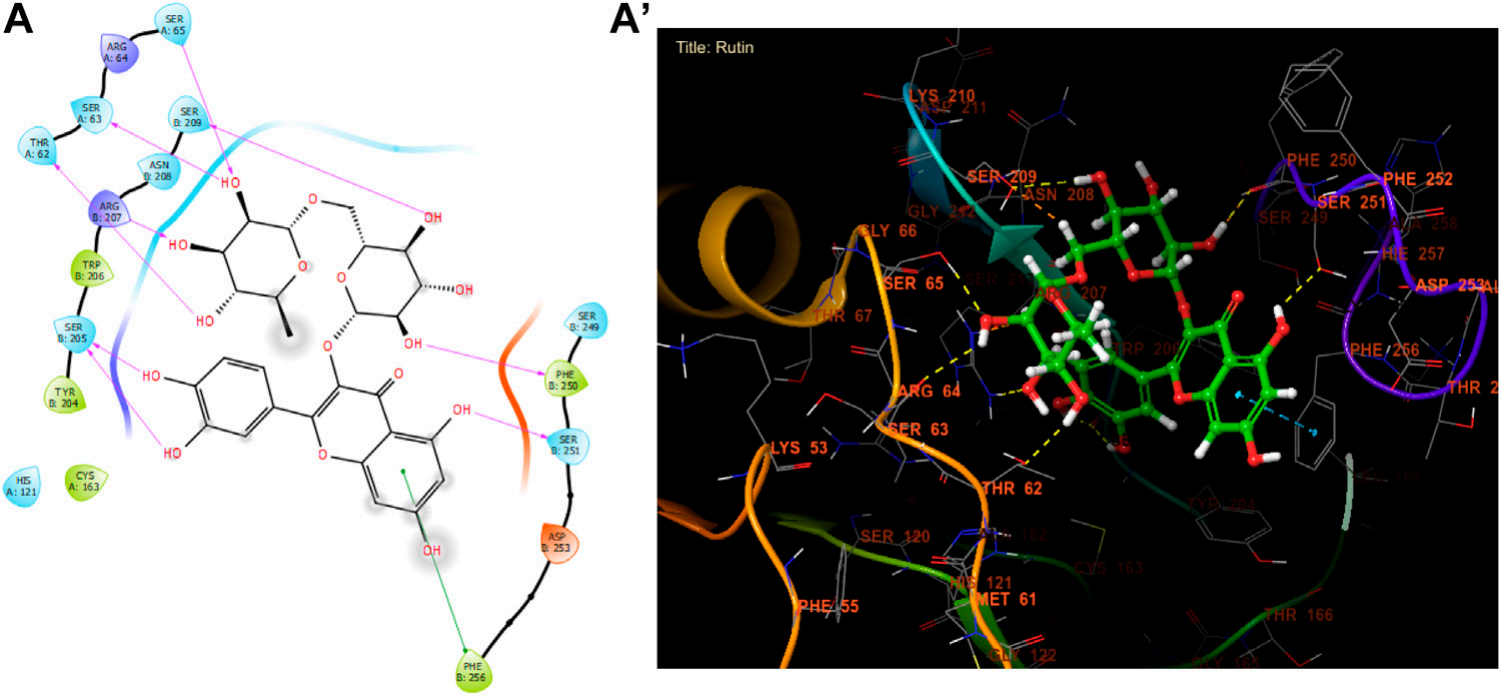
The ligand “Rutin” interacts with the active site of caspase-3 through the 2D viewer **(A)** and the 3D viewer **(A’)**.

In our *in silico* study, Rutin, Naringin, and Catechin were the most active molecules in the active site of caspase-3 with a glide gscore of −7.00, −6.54, and −6.51 kcal/mol (Table 10). Furthermore, Rutin established nine hydrogen bonds with the residues, SER A 65, SER A 63, THR A 62, SER B 209, ARG B 207, SER B 205, PHE B 250, and SER B 251, and a single Pi-Pi stacking bond with the PHE B 256 residue in the active site of caspase-3 (Figure 7).

**TABLE 10:** Glide gscore based on targeted caspase-3 protein and bioactive compounds of leaf extracts.

Caspase-3 (PDB ID: 3GJQ)	Leaf extracts bioactive compounds							
	Caffeic acid	Catechin	Kaempferol	Myricitin	Naringin	Rutin	Quercetin	Trans ferulic
Glide gscore (Kcal/mol)	−6.00	−6.51	−5.75	−5.80	−6.54	−7.00	−5.91	−6.07

## 4 Conclusion

According to the collected data, fig leaves are a sustainable source of industrially useful bioactive compounds with many potential applications. The analysis of hydro-ethanolic leaf extracts from fig trees in Eastern Morocco revealed a diverse range of bioactive compounds, particularly polyphenols and flavonoids. These compounds contribute to the extracts’ strong antioxidant capacity and antimicrobial activity against various bacterial and fungal strains. Specifically, the extracts demonstrated bactericidal effects against gram-positive strains (*S. aureus* and *B. subtilis subsp. spizenii*), gram-negative strains (*E. coli* and *P. aeruginosa*), and fungicidal effects against *C. albicans*. Additionally, the extracts exhibited significant cytotoxicity and a high selectivity index against three breast cancer cell lines: MCF-7, MDA-MB-231, and MDA-MB-436. Nevertheless, additional research, including *in vivo* tests, is necessary to validate these effects. Fig tree leaves have functional compounds and properties that make them suitable for use in the food industry, as they offer a variety of health benefits. Additionally, ongoing pharmaceutical research aims to identify new medications that exhibit improved efficacy and reduced adverse effects. *Ficus carica* is a plant with a variety of medicinal properties and potential therapeutic applications. Furthermore, their utilization could provide advantages to farmers by ensuring the sustainable administration of this waste. It is recommended to conduct further research on the incorporation of this leaf component and/or its extracts into food matrices for potential benefits.

## Data Availability

The original contributions presented in the study are included in the article/supplementary material, further inquiries can be directed to the corresponding authors.
